# Inflammation in ALS/FTD pathogenesis

**DOI:** 10.1007/s00401-018-1933-9

**Published:** 2018-11-21

**Authors:** Madelyn E. McCauley, Robert H. Baloh

**Affiliations:** 1 Board of Governors Regenerative Medicine Institute, Los Angeles, USA; 2 Department of Neurology, Center for Neural Science and Medicine, Cedars-Sinai Medical Center, 8700 Beverly Blvd, Los Angeles, CA 90048 USA

**Keywords:** Amyotrophic lateral sclerosis, Frontotemporal dementia, Neurodegeneration, Neuroinflammation

## Abstract

Amyotrophic lateral sclerosis (ALS) and frontotemporal dementia (FTD) are neurodegenerative diseases that overlap in their clinical presentation, pathology and genetics, and likely represent a spectrum of one underlying disease. In ALS/FTD patients, neuroinflammation characterized by innate immune responses of tissue-resident glial cells is uniformly present on end-stage pathology, and human imaging studies and rodent models support that neuroinflammation begins early in disease pathogenesis. Additionally, changes in circulating immune cell populations and cytokines are found in ALS/FTD patients, and there is evidence for an autoinflammatory state. However, despite the prominent role of neuro- and systemic inflammation in ALS/FTD, and experimental evidence in rodents that altering microglial function can mitigate pathology, therapeutic approaches to decrease inflammation have thus far failed to alter disease course in humans. Here, we review the characteristics of inflammation in ALS/FTD in both the nervous and peripheral immune systems. We further discuss evidence for direct influence on immune cell function by mutations in ALS/FTD genes including C9orf72, TBK1 and OPTN, and how this could lead to the altered innate immune system “tone” observed in these patients.

## Introduction

Amyotrophic lateral sclerosis (ALS) is a late-onset neurodegenerative disorder that primarily affects motor neurons. Loss of these neurons causes paralysis and death usually 3–5 years after symptom onset. The incidence of ALS in Europe and North America is reported to be between 1.5 and 3 per 100,000 people per year, with no treatments available that significantly alter disease course [65, 101]. Most cases of ALS are “sporadic” as they occur without a known cause or a family history. However, about 5–10% are caused by genetic mutations, typically passed down with dominant inheritance [22]. Neuropathological features include degeneration of motor neurons in the anterior horns of the spinal cord, brainstem, and large pyramidal neurons in the primary motor cortex. The death of motor neurons is accompanied by significant glial reaction, and ubiquitinated protein inclusions which typically contain the RNA-binding protein TDP-43. During the last few decades, many genes have been discovered that cause familial ALS or are over-represented in ALS patients compared to controls. These genes are involved in several cellular pathways, including RNA metabolism, protein homeostasis, and cytoskeletal dynamics, and provide researchers with tools to model ALS and study the molecular mechanisms underlying the disease. While all these genes are expressed in a variety of non-neuronal cells, including cells of the immune system, the majority of research has focused on their role in neurons, as they are the cells that ultimately degenerate in ALS.

ALS shares significant overlap with another fatal neurodegenerative disease, frontotemporal dementia (FTD) [111]. FTD is characterized by degeneration of the frontal and temporal lobes, and is associated with behavioral and personality changes, and impairment in social interactions. Approximately, 15% of patients with FTD develop motor neuron dysfunction, and up to 50% of patients with ALS develop frontal lobe dysfunction [88]. Pathologically, ALS and FTD are thought to be at different ends of a spectrum, with the presence of ubiquitinated neuronal cytoplasmic inclusions positive for TDP-43 found in both diseases. The two also share common genetic origins, with a mutation in C9orf72 being the most common cause [158]. Hexanucleotide repeat expansions in C9orf72 have been identified in up to 40% of familial ALS patients, and 20% of familial FTD patients, in addition to around 6% of sporadic ALS and FTD patients [34, 128]. Multiple other genes, such as VCP, FUS and TARDBP have also been linked to both diseases, and in each case members of a family can manifest either ALS, FTD, or both [77, 78, 84, 142, 154]. However, while the clinical, genetic and pathologic overlaps are well defined, the reasons why individual patients manifest different parts of the ALS/FTD spectrum remains a mystery.

Neuroinflammation is a term that broadly describes the reaction of resident glial cells (astrocytes, microglia) and circulating immune cells (monocytes, neutrophils, lymphocytes) that enter and interact with cells of the CNS in the context of infection, injury or degeneration. Neuroinflammation in ALS, as in other neurodegenerative diseases, is characterized primarily by an innate immune response rather than an adaptive immune response [126]. However, while astrocyte and microglial activation are the most prominent features on pathology, autopsy tissue from ALS patients also displays T cells [38, 79, 167], and non-resident innate immune cells (dendritic cells, macrophages, mast cells) [59, 68, 70]. The role of these other immune cells in disease pathogenesis remains poorly understood.

In addition to neuroinflammation, there is also evidence for systemic inflammation in ALS, as altered circulating lymphocyte and monocyte populations have been reported [107, 166], as well as inflammatory cytokines and other immune markers [99]. However, while neuroinflammation and systemic inflammation are uniformly present in ALS patients, it is still debated whether these phenomena are simply a consequence of disease, or instead play a contributory or even causative role.

In this review, we focus on the role of immune cells in ALS pathogenesis, discuss how their dysfunction influences neuroinflammation and systemic inflammation, and could contribute to disease risk or progression. In particular, we focus on a group of ALS genes including C9orf72, TBK1, and OPTN that have well-characterized roles regulating innate immune cell function. We discuss how mutations in these genes could alter innate immune “tone” in ALS patients, through their effect on myeloid-derived innate immune cell populations in the brain and the periphery.

### Diversity of myeloid populations that influence neuroinflammation

Myeloid cells are generally defined as those resulting from a myeloblast lineage, and include neutrophils, basophils, eosinophils, monocytes/macrophages and dendritic cells [48]. They originate from hematopoietic cells in the bone marrow and are constantly distributed throughout the body. They play a key role in the innate immune response, producing cytokines and chemokines to mitigate infection, activating the adaptive immune system through antigen presentation, and maintaining tissue homeostasis [48].

In the CNS parenchyma, microglia are the primary myeloid cell type, making up 5–15% of cells in the brain [91, 119] and are responsible for development, immune surveillance, and tissue homeostasis [83, 96, 134, 149]. During steady state, these cells are constantly surveying their environment, and upon injury microglia migrate to the damaged areas and produce cytokines and neurotrophic factors to mitigate damage [83]. Through phagocytosis, microglia engage in the clearance of pathogens and debris, as well as synaptic elements during development and likely disease [72]. As a stereotyped response to a pro-inflammatory environment, microglia also change their morphology and upregulate Iba1, CD11b and antigen presentation molecules (CD80/CD86) in response to pathogens or infection [89]. Microglia are unique among tissue macrophages in that they are a self-renewing population derived from early yolk-sac precursors, and are not replaced or expanded by circulating macrophages except in the case of tissue injury [2, 16, 55]. Under homeostatic conditions, there are few if any infiltrating myeloid cells in the CNS; however, breakdown of the blood–brain barrier in many disease states can lead to infiltration of monocyte-derived macrophages that are phenotypically different from resident microglia, but similarly work to alleviate damage caused by an insult [1, 62, 109, 123].

**Fig. 1:**
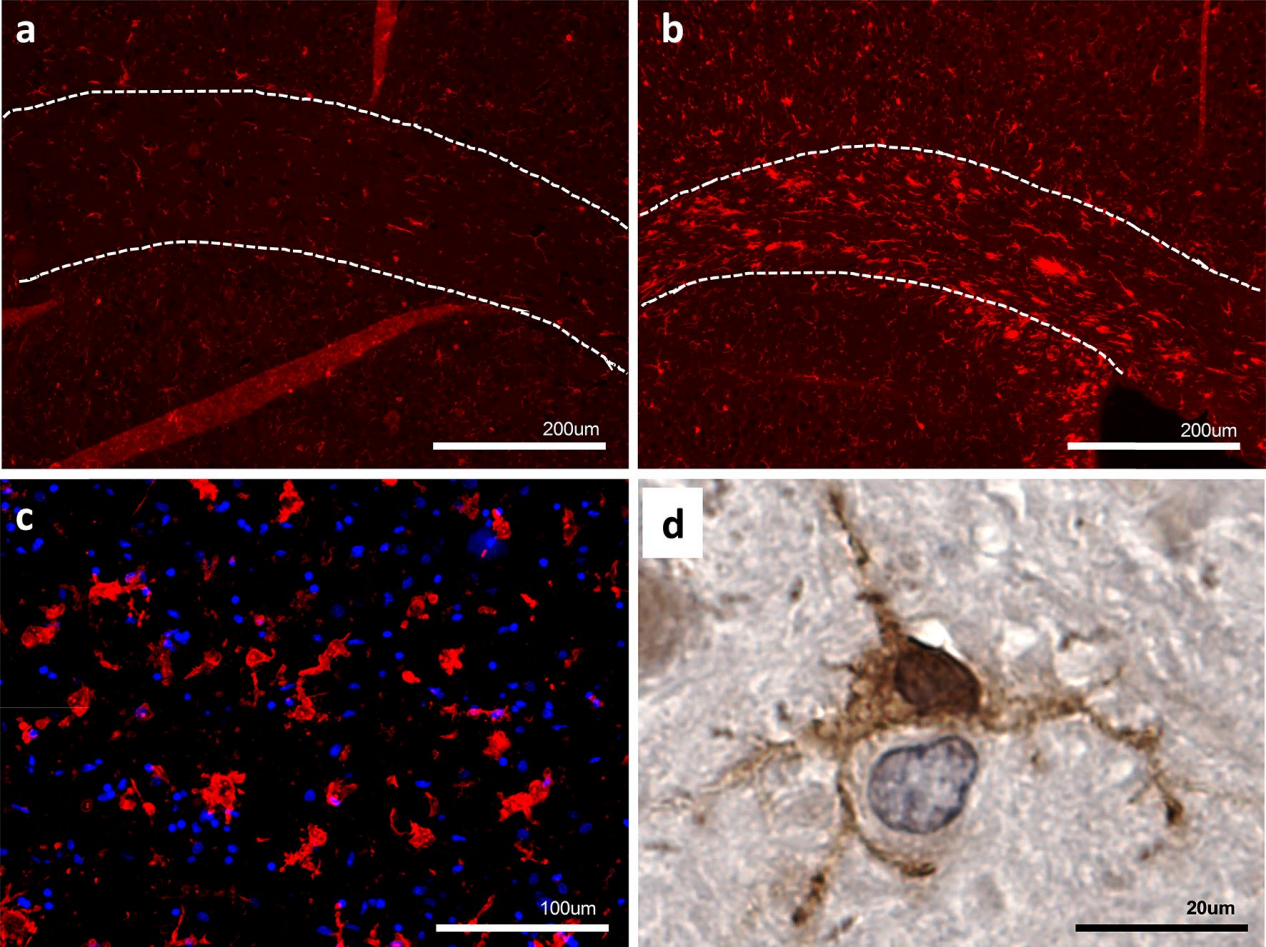
Microglial responses in neuronal injury and ALS/FTD. **a**, **b** Immunofluorescence image of Iba1 stain in the region of the motor cortex and corpus callosum (highlighted with dotted white lines) in the normal mouse brain (**a**), or after experimental traumatic brain injury (**b**). After tissue injury, Iba1-positive microglia proliferate and become activated with altered morphology, function and transcriptional profile to assist in clearing damage and maintaining tissue homeostasis. Scale bar 200 μm. **c** Microglial activation in motor cortex from a human subject with sporadic ALS (IBA1—red; DAPI—blue). Microglia appear similar on histology to tissue injury, with proliferation and activation characterized by enlarged ramifications. Scale bar 100 μm. **d** Iba1 (DAB stain) labeling of ventral horn of a mouse lacking the *C9orf72* gene, showing the interaction between the ramifications of a microglial cell and a neighboring neuron, can either be supportive or detrimental in the context of ALS/FTD as discussed in the text. Scale bar 20 μm

Microglia are extremely sensitive to changes in their environment and are known to react to danger signals present both within the CNS and systemically (Fig. 1). Unregulated activation of microglia leads to the production of potentially harmful neurotoxins, such as reactive oxygen species (ROS), quinolinic acid and reactive nitrogen species (RNS), which can be detrimental to neighboring neurons [160, 170]. As noted above, microglia can take on either a damaging or protective phenotype depending on the context of their activation, and early studies suggested that microglia assume finite activation states that correlate with these two activities [127]. Due to recent advances in single-cell sequencing, the concept that microglia exist in only two different activation states has been demonstrated to be an over-simplification. As an example, using transcriptional single-cell sorting all immune cells in the brain of wild type and a transgenic mouse model of Alzheimer’s disease were mapped, identifying numerous states including a population called disease-associated microglia (DAM) [81]. Another study used single-cell RNAseq to examine multiple distinct reactive microglia populations in the hippocampus of AD mice [97]. This study looked specifically at progression of neurodegeneration at multiple time points, and interestingly they identified distinct reactive microglia phenotypes, characterized by the production of type I and type II interferon genes [97]. These microglia were different from the previously identified DAM, in that they expressed interferon-related or proliferation molecules. Additional diversity of microglial responses was recently reported from whole-tissue RNA profiles from human neurodegenerative diseases [47]. These studies have just begun to clarify the underappreciated heterogeneity in how microglia respond to environments such as neurodegeneration, and alter the way researchers approach characterizing and therapeutically targeting these cells.

#### Other CNS-resident macrophages

In addition to microglia, there are several other CNS-resident macrophages present throughout the brain and spinal cord that could respond to and incite systemic inflammation, and influence brain function. They consist of perivascular macrophages (PVM), meningeal macrophages and choroid plexus macrophages [58, 82]. These cells produce copious amounts of ROS, alter the neurovasculature of the CNS, recruit circulating immune cells, and incite systemic inflammation [40, 41, 66]. Studies suggest these other myeloid populations may play a role in neurodegenerative diseases such as Alzheimer’s, as well as experimental autoimmune encephalomyelitis (EAE) [118, 122]. For example, PVMs are believed to be involved in waste product clearance. In a model of AD, depleting PVMs had no effect on amyloid plaques in the brain parenchyma, but there was an increase of amyloid beta around the cerebral blood vessels [118]. In EAE, the number of PVM increases after onset of disease and depleting these cells ameliorated neurological symptoms [122]. While not fully understood, it could be that PVMs are acting as antigen-presenting cells that are able to reactivate T cells as they cross the blood–brain barrier [122]. Although not yet studied in animal models of ALS, these additional myeloid cells may play a role in neuro- and systemic inflammation in ALS pathogenesis, warranting further investigation.

### Neuroinflammation in ALS/FTD—pathology, imaging and model systems

Most of the earliest observations regarding inflammatory changes in ALS come from the study of patient autopsy material. The key pathologic features of ALS include ubiquitinated neuronal inclusions typically positive for the RNA-binding protein TDP-43, and motor neuron and interneuron cell loss in the cortex and spinal cord, with adjacent enlargement and proliferation of microglia and astrocytes typically referred to as “activated” [79]. The activation of microglia and astrocytes in ALS occurs with minimal infiltration of peripheral immune cells, which is a characteristic of the pathology of most neurodegenerative diseases, supporting that innate immune sensing pathways are most prominent and activated earliest [126]. The activation state of astrocytes and microglia has been traditionally observed by immunostaining for glial fibrillary acidic protein (GFAP) for astrocytes, and IBA1 or CD68 for microglia, and the degree of microglial pathology correlates with rate of disease progression [21]. However, as described above recent studies of diversity in microglial and astrocyte responsiveness indicate that these markers do not adequately represent the range of molecular response to tissue injury generated by these cells [18]. Additionally, because autopsy tissue is obtained at disease end stage and after a period of post-mortem ischemia, it is difficult to determine if the changes observed only occur late in disease progression. While a variety of peripherally derived inflammatory cell types have also been observed in areas of cellular injury in ALS, including T cells, mast cells, monocyte-derived macrophages and dendritic cells, these remain a minor component of the lesions compared to their frequency in inflammatory or infectious brain diseases, leaving their relevance unclear [38, 59, 70, 132].

Although autopsy studies reflect events late in disease, data from human imaging studies support that glial activation begins early in disease pathogenesis. Studies using [11C](R)-PK11195 to label “peripheral benzodiazepine-binding site”, expressed by activated microglia, first showed that increased binding was present in affected areas of brain in ALS patients with a range of severity and disease duration, and motor cortex signal correlated with the degree of clinical upper motor neuron signs [150]. Subsequently, the use of PET ligands that bind to the 18pkD translocator protein (TSPO) expressed in activated microglia and astrocytes demonstrated binding in affected brain regions as early as the time of diagnosis [33]. A recent study using another TSPO binding ligand PET integrated with MRI showed that areas of ligand binding correlated with cortical thinning, reduced fractional anisotropy, and increased diffusivity, in addition to the clinical upper motor neuron score [5]. Interestingly this study did not see a change in ligand binding in subjects undergoing repeat imaging over 6 months despite clinical progression, suggesting glial activation is present early and does not change at least in terms of TSPO imaging. Neuroinflammation is also a key component of FTD, supported by similar studies showing increased TPSO binding in several affected brain regions, including the cortical frontal, mesial temporal, subcortical regions, prefrontal cortex, hippocampus and parahippocampus in patients compared to controls [26]. Additionally, FTD patients have increased levels of cytokines in their CSF compared to healthy controls, supporting that there is activation of the intrathecal immune response as in ALS [139].

Rodent model systems have the advantage of allowing researchers to investigate tissue changes that occur before end stage of disease. In the case of ALS, studies of rodent models have supported the idea that neuroinflammation begins very early in disease course. In the widely studied SOD1 mouse model, activated microglia are present near motor neurons before the onset of weakness, and the degree of inflammatory response correlates with disease progression [76, 87, 132]. T cells are also found in the spinal cord, but this seems to occur later after microglial activation [4]. Microglia have been shown to increase their production of pro-inflammatory cytokines, TNFα and IL1β, as well as generate an excess of reactive oxygen species (ROS), which could potentially directly harm neighboring motor neurons suggesting this early glial reaction could actively be damaging neurons [69]. Multiple lines of experimentation from the SOD1 model support that on the whole, microglial activity is detrimental when considered across the full duration of disease [12, 19, 87]. However, it is important to note that the visual presence of “activated” microglia on histology does not indicate they are damaging motor neurons, and there is evidence that they may instead be promoting neuronal health or recovery. For example, in the SOD1 mouse model, microglia have been demonstrated to shift from a neuroprotective to a neurotoxic phenotype over the course of disease [12, 87]. Recently, another group demonstrated in a TDP-43 transgenic model that microglia initially showed minimal reaction to neuronal pathology, but after the transgene was suppressed, microglial “activation” occurred which correlated with promotion of recovery instead of injury [141]. Animal models likewise support the occurrence of neuroinflammation and microglial activation in FTD. In particular, knockout models of progranulin (Grn) show increased astrogliosis and microgliosis in the hippocampus, cortex and thalamus [51, 164]. Interestingly, reducing Grn levels in only neurons or microglia does not result in neuroinflammation measured by Iba1 and GFAP staining, suggesting the inflammation is driven non-cell autonomously [121].

In summary, neuroinflammation in ALS/FTD is primarily characterized by activation of innate immune sensing pathways in microglia and astrocytes resident to the CNS. The bulk of evidence from imaging studies in humans, and pathology studies in model organisms support that this process occurs early, long before neuronal cell death, via yet unknown signaling events between injured neurons and neighboring glial cells. The first cells to respond are likely microglia, as they are exquisitely sensitive to any perturbation in their environment. However, the exact signaling molecules and cellular states that are induced by these molecules need to be more clearly defined, as the term “activation” is used in the literature to describe glia that appear similar on pathology, but can either promote a toxic or restorative environment for neurons. Therefore, more studies and better tools are needed to clearly define how neuronal-glial signaling occur at different stages of disease, and where potential therapeutic interventions can be made to modulate inflammation to slow disease progression.

### Alterations in systemic immune markers in ALS/FTD patients

During a localized infection or injury, a systemic immune response frequently occurs leading to production of cytokines and mobilization/activation of circulating immune cells to help mitigate tissue damage and restore homeostasis. For this process to be effective, checks and balances (anti-inflammatory cytokines and immune cells) need to be in place to dampen the systemic inflammatory response appropriately and maintain immune tolerance to self-antigens. Because these processes are occurring simultaneously, studies profiling immune markers in peripheral blood are sometimes challenging to interpret, as they are dependent on a complex mixture of disease state, recent environmental exposures, and genetic background of the subject. Despite these challenges, a detailed understanding of the systemic immune response in the context of ALS and FTD is not only important to unravel disease mechanism, but potentially to serve as a biomarker of disease activity.

Blood from ALS patients have consistently shown changes in systemic inflammatory markers and immune cell populations compared to healthy controls, with ALS patients having differences in levels of neutrophils, CD4 and CD8 lymphocytes and CD16 monocytes, with CD16 monocyte levels correlating with disease severity [57, 107, 166, 171]. Levels of circulating cytokines are also abnormal in ALS patients, with altered production of IFNγ, IL-2, IL-8, IL12p70, TNFα, IL-1b, CK, ferritin, IL-4, IL-5, IL-10 and IL-13 [90]. Having increased levels of ferritin and IL-2 correlated with poorer survival probability suggesting a role of the peripheral immune system in disease progression [90]. FTD patients have also been reported to have elevated levels of circulating cytokines; however, due to partially conflicting findings, further studies are warranted [20, 52].

In addition to cytokine production, and lymphocyte and monocyte populations, alterations in dendritic cells have been observed in ALS [70]. Dendritic cells (DCs) are innate immune cells adept at cytokine production and antigen presentation, and play a key role in regulating the adaptive immune system [159]. mRNA expression of DC surface markers in both sporadic ALS and familial ALS shows immature and activated/mature DC transcripts which are significantly upregulated in ALS tissues, and immunohistochemistry confirmed the presence of these cells in the ventral horn and corticospinal tracts [70]. In addition, the chemokine MCP-1, which recruits monocytes, memory T cells and DCs to sites of inflammation, was found to be expressed by glia and in the CSF from ALS patients, but not control subjects. Interestingly, patients who progressed rapidly had significantly more dendritic cell transcripts than patients who slowly progressed, implicating peripheral immune cell recruitment in disease pathogenesis [70].

Although a systemic immune response is essential to fight infection, it can be detrimental to the whole organism if it is not appropriately regulated. Regulatory T lymphocytes (Tregs) are a key cell type responsible for suppressing the immune response and maintaining immune tolerance [130]. Tregs maintain homeostasis by inhibiting effector T-cell proliferation and cytokine production, shutting down an overactive immune response. In accord with the pro-inflammatory state reported in peripheral blood from ALS patients, fewer Tregs have also been observed in patient and rodent models of ALS [13, 14, 93, 136]. Treg levels were found to correlate with disease progression rates in mutant SOD1 mice, and passive transfer of Tregs suppressed neuroinflammation and prolonged survival in these animals [169]. Likewise, rapidly progressing patients were found to have decreased numbers of Tregs and FOXP3 protein expression, a key transcription factor crucial for development and function of Tregs [68]. Given the correlation between Treg levels and disease progression, a phase 1 trial was recently performed to infuse ex vivo-expanded autologous Tregs into ALS patients, and it appeared overall safe and well tolerated [147].

In summary, a variety of systemic inflammatory responses (pro- and anti-inflammatory cytokine profiles, altered immune cell populations) have been consistently reported in ALS patients. This could be driven by several factors including: i) a response to tissue damage in the brain, initially sensed by resident glia then amplified and propagated by the peripheral immune cells; ii) an intrinsically altered peripheral immune system in ALS and FTD patients driven by genetic differences. The idea that the immune system is intrinsically altered in ALS and FTD patients is becoming of increasing interest given that mutations in several ALS/FTD genes have been shown to have a significant direct effect on immune cell function (see below).

### Associations with autoimmunity and cancer risk suggest altered innate immune system “tone” in ALS/FTD patients

Dating back to the 1980’s researchers noticed patients with ALS have a higher than expected incidence of autoantibody production, and monoclonal or polyclonal gammopathy [37, 116, 138]. The initial assumption was that these antibodies may mediate an autoimmune attack on motor neurons [35]; however, the lack of response of ALS patients to plasma exchange or other antibody-directed therapies or immunomodulatory therapies argued against this idea. Likewise, no individual autoantibody has thus far been discovered which could explain disease pathophysiology, and instead ALS patients have been found to generate a wide variety of antibodies to autoantigens, most of which do not appear to have functional significance. These include antibodies against ganglioside GM1 and GD1a [120], neurofilament proteins and sulfoglucuronylparagloboside [15], FAS (CD95) [163], and voltage-gated Ca2+ channels (VGCC) [7, 152]. More recent studies support that ALS patients can be readily distinguished from controls by the presence of a large panel of autoreactive IgG antibodies [98]. While the lack of identification of a specific pathogenic autoantibody or response to antibody-targeted therapies initially lowered the enthusiasm for these findings, they strongly support the idea that ALS patients have an altered peripheral immune system, one that has the tendency to break tolerance and generate a variety of autoantibodies.

Recent epidemiological studies have further supported the idea that ALS and FTD patients have an intrinsically altered immune system, finding that significantly more cases than expected of ALS are associated with a prior diagnosis of an autoimmune disorder such as asthma, celiac disease, systemic lupus erythematosus, ulcerative colitis, myasthenia gravis and juvenile-onset diabetes [151]. A similar increased risk of autoimmune disorders was also observed in a cohort of FTD patients with TDP-43 pathology, and subsequently in a cohort of specifically C9orf72 gene mutation carriers, compared to normal controls or subjects with other neurodegenerative diseases [103, 104]. Further connecting C9orf72 expansion carriers to autoimmune disease, in a small cohort of patients diagnosed with the rare combination of multiple sclerosis and ALS, a remarkable 80% carried the hexanucleotide repeat expansion in C9orf72 [73]. These data support that ALS/FTD patients have a tendency toward developing autoimmune disorders, which could be driven by specific gene variants in these patients, including C9orf72 repeat expansion.

Despite evidence supporting a connection between ALS/FTD and risk of autoimmunity, there is little evidence that ALS is itself an autoimmune disease, as traditional medications to suppress the immune system failed to slow disease progression including corticosteroids, azathioprine and cyclophosphamide [8, 11, 23, 36, 105]. Plasmapheresis and intravenous immunoglobulins also failed to alter disease progression supporting that the autoantibodies observed in the disorder are not pathogenic [80, 105, 115]. The most likely reason behind the lack of response in these trials is that the interventions did not alter inflammatory events taking place in the nervous system, and that the tendency toward autoimmunity and autoinflammation in ALS/FTD patients is driven by a shared cause, i.e. environmental or genetic lesions that promote a tendency to develop either ALS/FTD, autoimmunity, or sometimes both.

The idea that the immune system in ALS/FTD patients is shifted toward a pro-inflammatory tone raises several interesting questions. It is becoming well established that cancer immunity and autoimmunity are two sides of the same coin, with checkpoint inhibitor therapy leading to both the promotion of tumor immunity as well as autoimmunity [148]. Antigen-presenting cells, such as dendritic cells, play a key role in driving the innate immune system tone and balancing tumor immunity vs. autoimmunity by the adaptive immune system [32, 49]. Therefore, one would predict based on the tendency toward autoimmunity, that ALS/FTD patients may have a decreased incidence of cancer. Interestingly, a recent study did observe that overall risk of cancer at any site was found to be significantly reduced in ALS patients [53]. It is important, however, to note that earlier studies showed conflicting results as to the relationship between cancer and ALS [39, 45], so further studies are needed to determine the nature and degree of this effect.

### How mutations in ALS/FTD-associated genes could directly influence the innate immune system

Over the last 10 years, many ALS/FTD-associated genes have been discovered, nearly all of which are ubiquitously expressed [17]. Interestingly, some of these genes (PGRN, C9orf72) are more highly expressed in non-neuronal cells, including microglia, than they are in neurons. Others (TBK1, OPTN, SQSTM1) have been extensively studied regarding their role in regulating the function of innate immune cells. This raises the possibility that ALS and FTD mutations could directly influence the function of immune cells, and perhaps act in concert with the effect of these mutations on neurons to drive disease. We review below the ways in which several ALS/FTD genes have been shown to impact immune cell function directly.

#### SOD1

Mutations in SOD1 were one of the first major ALS genes identified and make up for ~ 15% of familial ALS. Transgenic mice overexpressing human mutant SOD1 (mSOD1) are the most commonly used model to study disease pathogenesis, as they reliably develop a mutation-dependent fatal motor neuron disease [24, 60, 157]. However, while the phenotype is driven by spinal motor neuron loss, the toxicity of mSOD1 is not cell autonomous to motor neurons. Early studies showed that overexpressing mSOD1 in neurons alone for up to 1.5 years did not result in motor deficit [125, 162], supporting that non-neuronal cells contributed to the motor neuron loss. Other studies showed that relative to wild-type microglia, mSOD1 microglia produced more superoxide, nitric oxide and TNFα when stimulated with LPS compared to wild-type microglia [156, 160, 170]. These mSOD1 microglia caused more injury to primary cultured motor neurons compared to wild-type microglia [168], and inhibiting production of NF-kB was found to suppress mSOD1 microglia toxicity when co-cultured with motor neurons [44]. Using mice with chimeric mosaicism to express mSOD1 in different cell populations, it was demonstrated in vivo that wild-type motor neurons surrounded by mSOD1-expressing glial cells developed features of ALS pathology, while mSOD1-expressing neurons surrounded by healthy glia remained disease free [31]. Finally, Cre–lox-driven removal of mSOD1 from microglia led to a longer lifespan despite the persistent expression of mSOD1 in motor neurons [19]. Together, these studies across in vitro and in vivo platforms show that intrinsic changes in microglia from mSOD1 expression directly contribute to motor neuron degeneration in the mSOD1 mouse model.

As discussed earlier, the early onset of the microglial activation in mSOD1 mice support that mSOD1-expressing microglia could directly damage motor neurons and promote disease progression [4, 61]. As a potential demonstration of this, mSOD1 was shown to activate caspase-1 and IL1β in microglia (independent of their activation by environmental cues) and preventing the production of IL-1β-attenuated inflammation and enhanced survival in mSOD1 animals [102]. These examples from SOD1, the longest established ALS-associated gene, provide strong evidence for a cell intrinsic effect of genetic mutations on microglia, and set the premise for considering whether more recently discovered ALS and FTD genes may also similarly alter myeloid cell function to impact neurodegeneration non-cell autonomously.

#### C9orf72

Expansions in a hexanucleotide repeat (GGGGCC) in a noncoding region of the C9orf72 gene are the most common cause of familial and sporadic ALS and FTD to date, accounting for roughly 40% of familial ALS and 5–10% sporadic ALS [34, 128]. Healthy individuals contain 2–20 repeats, while affected individuals typically have hundreds or thousands. Highlighting a potential role in the immune system, C9orf72 is highly expressed in many myeloid cell types, including microglia and circulating monocytes [67, 108, 112, 129].

There are currently three main hypotheses as to how C9orf72 repeat expansion causes disease. First, as usage of the upstream 1a promoter leads to transcription of the repeat, RNA-mediated toxicity has been proposed due to the presence of sense and antisense RNA foci in cells and autopsy tissue from C9orf72 patients. These foci could sequester RNA-binding proteins and alter RNA metabolism in patients [34, 50, 85]. Second, simple poly-dipeptides produced by repeat-associated non-AUG (RAN) translation of the repeat containing RNA have been found to accumulate in the brain and spinal cord of C9orf72 mutation carriers, which have a variety of toxic properties [9, 50, 92]. Third, loss of function of the C9orf72 gene product could contribute, as the presence of the repeat expansion leads to downregulation of C9orf72 expression (particularly from the 1b promoter) [133]. Decreased transcript levels have consistently been demonstrated from C9orf72 patient brain tissue [34, 54]. Further supporting this idea, studies looking at *C. elegans* and zebrafish have shown that complete loss of C9orf72 leads to motor neuron degeneration [30, 146]; however, similar findings have not been observed in mammals as discussed below. Studies thus far examining the molecular and cellular biology of C9orf72 indicate that it regulates endosomal trafficking, including autophagy [42, 135, 144] and lysosomal function [6, 112].

Human genetic studies strongly support that ALS/FTD cannot be haploinsufficient of C9orf72 alone, as patients with premature stop codons would almost certainly have been identified through existing sequencing projects for ALS and FTD [63]. However, there is strong data supporting that loss of function could contribute to disease. A recent case study reported a 90-year-old C9orf72 carrier who passed away from unrelated causes, and his ALS-affected child. Both individuals harbored the pathogenic repeat, but the father was a mosaic with a small expansion in blood (~ 70) and large expansions in CNS tissues. On pathology, the unaffected father and affected daughter both had equivalent RNA foci and dipeptide repeat pathology. However, the C9orf72 expression levels were significantly higher in the father compared to his daughter, presumably because of the mosaicism of his repeat [100]. This suggested the presence of RNA foci and DPR pathology was insufficient to promote neurodegeneration, and that decreased levels of C9orf72 needed to also be present. In accord with this, a recent study of iPSC-derived motor neurons from C9orf72 patients supported that haploinsufficiency of the protein was the primary driver of the survival phenotype in these cells, rather than toxic dipeptide repeats or RNA foci production [137].

Several groups developed knockout mice to study how loss of C9orf72 could play a role in neurodegeneration. Animals lacking one or both copies of C9orf72 never developed signs of neurodegeneration or motor system dysfunction. Instead, these animals developed progressive splenomegaly and lymphadenopathy, increased production of pro-inflammatory cytokines, altered immune cell populations, autoinflammation with autoantibody production, and in some colonies autoimmune-like disease [10, 25, 112, 143]. While the core features of systemic inflammation and lymphoid tissue hyperplasia were consistent across all studies, there were environment-dependent differences in whether animals developed spontaneous and fatal autoimmune disease [25], autoantibody production with a lupus nephritis-like syndrome [10], or normal survival with late-onset milder systemic and neuroinflammation [112]. Presumably, this relates to the different housing conditions in the different mouse facilities (pathogens, microbiome, etc.) but the exact drivers remain unknown. Importantly, the groups observed partial phenotypes in mice lacking one copy of C9orf72 in both cultured macrophages [112], and in immunoprofiling and survival [25]. These findings suggest that haploinsufficiency of C9orf72 is enough to drive an altered myeloid cell function and systemic immune response, which has important implications with C9orf72-associated ALS, as patients carry only one expansion allele and, therefore, show only partial loss of C9orf72 expression.

While it remains to be defined exactly which peripheral immune cells contribute to the systemic inflammation phenotype, it is clear that myeloid cells express relatively high levels of C9orf72, and show defects in lysosomal morphology and hyperactive responses to immune stimuli [112]. Brain microglia from C9orf72-deficient mice also showed lysosomal accumulations and increased production of pro-inflammatory cytokines, supporting that they could function abnormally in C9orf72 patients. Interestingly, transcriptional profiling of C9orf72-deficient spinal cords revealed age-related upregulation of inflammatory pathways, which overlapped with C9-associated FTD patient tissue more than sporadic FTD tissue, suggesting that altered or enhanced neuroinflammation may also exist in C9orf72-carrier brain tissue [21, 112].

While the finding of an immunologic phenotype and altered myeloid cell function from the loss of C9orf72 may have initially been surprising, it is interesting to note that similar phenotypes have been observed due to loss of function of other ALS/FTD-associated genes, suggesting that there may be a subgroup of these genes which similarly influence immune function.

#### TBK1

Mutations in the gene tumor necrosis factor receptor-associated factor NF-kB activator (TANK)-binding kinase 1 (TBK1) were recently identified as another genetic cause of ALS and FTD [29]. The large number of premature stop codons is most consistent with a loss of function and haploinsufficiency mechanism [46, 113, 124]. TBK1 is a ubiquitously expressed serine–threonine kinase, and was already widely studied for its role in regulating type 1 interferon (IFN) production [43]. Interestingly, loss of function mutations were previously reported in humans as causing susceptibility for childhood herpes simplex encephalitis, presumably because they led to a deficiency in type 1 IFN response [71]. TBK1 regulates numerous cellular pathways including IRF3 phosphorylation and IFN induction following infection, and autophagy through phosphorylation of OPTN, p62/SQSTM1 and SMCR8 (a C9orf72-binding partner), as well as influencing cell proliferation and growth [43, 94]. Loss of function mutations in TBK1 have subsequently been confirmed in studies of both familial and sporadic ALS and FTD, and are responsible for 1–2% of familial ALS [17, 29, 46, 124, 153]. The role for this protein in cell types of the CNS are only beginning to be discovered; however, there are some striking similarities in the effects of loss of TBK1 function in myeloid cells to those described for loss of C9orf72 above.

A recent study reported the conditional deletion of TBK1 in dendritic cells using CD11c-Cre mice [161]. Interestingly, knocking out TBK1 in this myeloid cell population led to progressive splenomegaly and lymphadenopathy similar to that seen in C9orf72-deficient mice. Dendritic cells lacking TBK1 also showed co-stimulatory molecule upregulation, and promoted an increased activation of CD4 and CD8 T cells. This heightened inflammatory state led to the animals having enhanced susceptibility to EAE, which is not surprising considering the role of dendritic cells in regulating the immune system and autoimmunity. This altered innate immune system tone also led to enhanced antitumor immunity, demonstrated by the increased survival rate and decreased tumor volume observed after inoculation with a melanoma cell line.

Interestingly, while type 1 IFN production by dendritic cells themselves was attenuated, the broader interferon response was hyperactive in these animals and appeared to drive much of the phenotype, as crossing CD11c-Cre TBK1 mice to interferon alpha receptor 1 (IFNAR1)-KO mice essentially reversed the splenomegaly, lymphadenopathy and T-cell activation states [161]. This apparent paradox may be explained by the fact they observed that TBK1 mediates phosphorylation of STAT3 serine which negatively regulates STAT1 activation, subsequently shutting down type 1 IFN production in a feedback loop [75, 155]. STAT3 is essential for the tolerant function of dendritic cells, and when ablated causes an increase in T-cell activation as well as susceptibility to autoimmunity [27]. This study highlighted a key role for the dendritic cell-specific function of TBK1 in the maintenance of immune homeostasis and tolerance, providing evidence of an immunoregulatory function as well as the previously known role in antiviral innate immunity.

Another study showed that lack of TBK1 in T cells led to an increase in the number of activated CD4 and CD8 T cells in the spleen. Interestingly, when challenged with the EAE model, ablating TBK1 in T cells led to retention of effector T cells in the draining lymph nodes, resulting in decreased numbers of T cells infiltrating into the brain, and a less severe disease phenotype [165]. This study suggests that in addition to the role TBK1 plays in innate immune cells, it may also function in lymphocytes, widening the scope of how this gene could contribute to inflammation in ALS. While further studies are needed, it is likely that alterations in systemic immunity and microglial function will be present in ALS/FTD patients with loss of TBK1 function, and raises the possibility that like for other ALS/FTD genes, altered function of immune cells will contribute to disease pathogenesis.

#### OPTN

Another ALS/FTD gene with previously identified roles in regulating immune cell function is optineurin (OPTN). Currently, more than 20 ALS-linked missense OPTN variants have been reported; however, most of them are still lacking in vitro/in vivo models [95]. Several studies have shown this gene plays a key role in mediating inflammation, and interestingly OPTN is a binding partner of TBK1 [56]. In both sporadic and familial ALS patients harboring the OPTN mutations, NK-kB immunoreactivity in microglia is increased compared to controls [131]. Other studies have shown that OPTN deficiency contributes to neuronal cell death via increased NF-kB activity. In addition, overexpression of wild-type OPTN was able to rescue induced cell death, while ALS mutant OPTN was not [3].

In addition to NF-kB regulation, OPTN has been implicated in necroptosis, a programmed form of necrosis linked to inflammation. Unlike apoptosis, necroptosis is caspase independent and can be triggered via TNFα. A recent study found that a key role of OPTN is RIPK-1-mediated-necroptosis [74]. In OPTN-deficient mice, they found that RIPK-1, RIPK-3 and MLK levels were increased in the spinal cord, and enhanced cell death and swelling of motor axons upon TNF treatment [74]. Conditional removal of OPTN in different cell types led to axonal pathology and myelin abnormalities; however, only when it was removed from oligodendrocyte or microglia, with no phenotype observed following motor neuron or astrocyte depletion [74]. Furthermore, OPTN depletion in microglia only led to axonopathy, suggesting a myeloid cell loss of the gene can drive neuronal pathology.

Like TBK1 and C9orf72, OPTN has also been shown to be involved in antiviral and antibacterial responses outside of the CNS. Overexpression of OPTN inhibits IL-1β and lipopolysaccharide (LPS)-induced NF-kB activation, while depleting OPTN resulted in increased LPS-induced NF-kB activation [106, 114, 145]. Other studies implicate OPTN in mediating the immune response to Salmonella infection [28, 140]. These findings indicate that the normal function of optineurin is essential to mediate the peripheral innate immune response, and support the idea that ALS-associated mutations may lead to changes in systemic and neuroinflammatory responses that could influence disease onset or progression.

#### TDP-43

One of the hallmarks of ALS and FTD is the presence of ubiquitinated inclusions in surviving neurons [86]. Transactive response DNA-binding protein-43 (TDP-43) is a multifunctional nucleic acid-binding protein that was first identified as the key component of these inclusions in ALS and FTD [110]. Subsequently, mutations in the glycine-rich C-terminal domain of TARDBP in sporadic and familial ALS cases were identified and established a causal link between TDP-43 and disease through as yet unclear mechanisms [64].

While the focus has primarily been on the role of TDP-43 aggregation in neurons, several studies support that non-cell autonomous neuronal-glia signaling could also contribute to neurodegeneration resulting from TDP-43 mutations. The most definitive example of this was provided recently when TDP-43 was conditionally deleted from microglia [117]. While the animals did not have a significant phenotype at baseline, microglia showed improved clearance of amyloid-β injected into the cortex, and there was enhanced engagement of microglia surrounding the injected particles. Likewise when mice lacking TDP-43 in microglia were crossed to an AD genetic model, they observed a significant decrease in the Aβ levels and aggregation, indicating that the loss of TDP-43 had driven a pro-inflammatory phenotype in the cells that improved their ability to clear plaques. Of note, while microglia depleted of TDP-43 showed an improved ability to clear plaques, they also had loss of synapses, which could potentially have a detrimental effect on disease progression [117]. These data support that as with other ALS/FTD disease genes, mutations in TDP-43 could have a direct and profound effect on microglial function and peripheral immune function, although the latter remains to be investigated.

## Conclusion

**Fig. 2:**
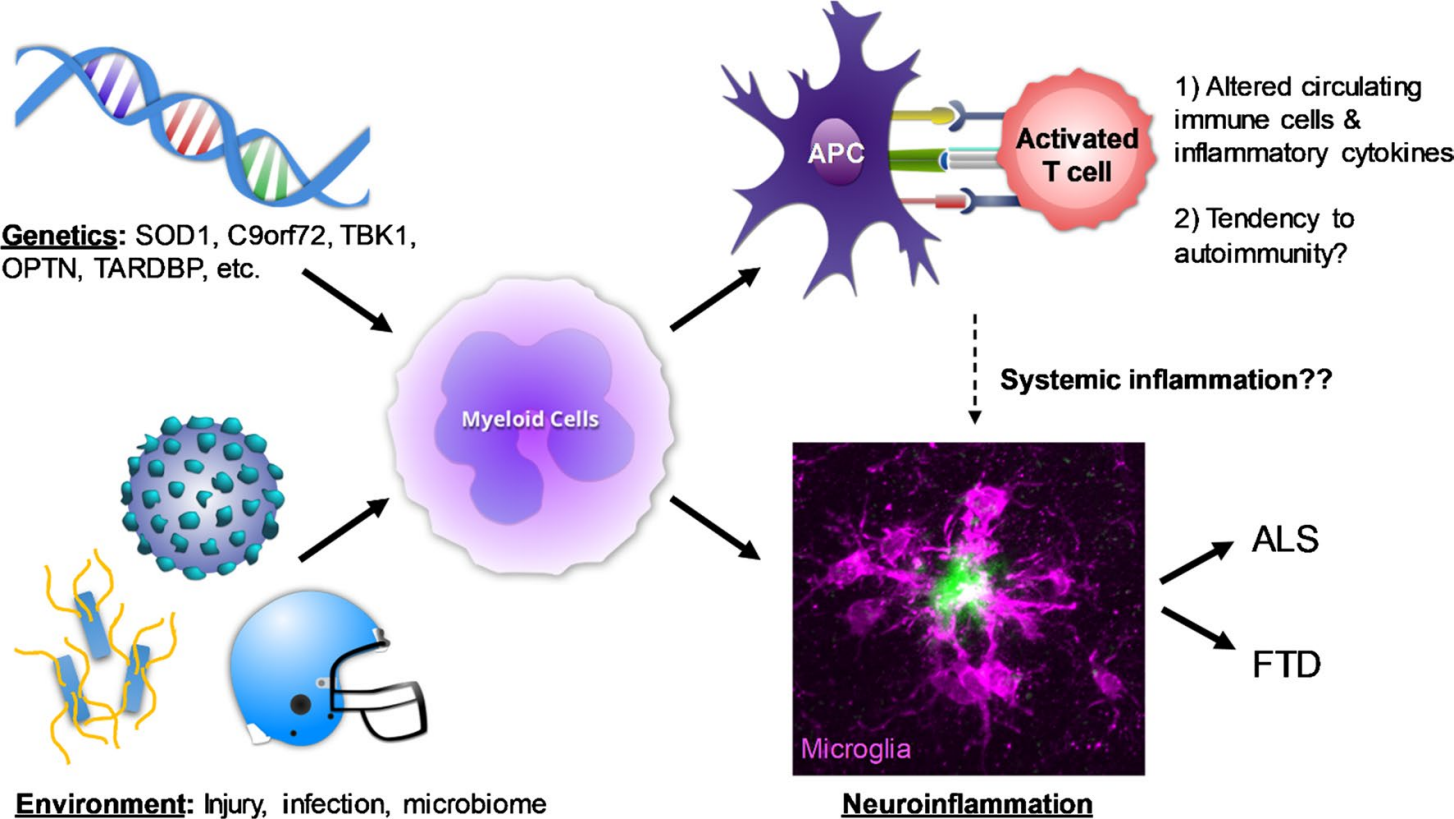
Schematic of potential interactions between environment, genetics and inflammation in ALS/FTD pathogenesis. Mutations in several ALS/FTD genes can directly disrupt the function of myeloid cells in different compartments and their response to environmental exposures. Altered function of peripheral myeloid-derived antigen-presenting cells (APCs) can promote activation of T cells and B cells, and generate an autoinflammatory state including the production of pro-inflammatory cytokines and autoantibodies, findings that have been observed in patients with ALS. The same genetic mutations can also cause microglial dysfunction, interacting with environmental exposures to drive the pathogenesis of degenerative brain diseases such as ALS/FTD. Finally changes in systemic inflammation can influence the function of microglia and neurons indirectly, and contribute to neurodegeneration. The photomicrograph in the bottom right shows microglia (purple, stained for IBA1) surrounding an amyloid plaque (green, stained for ThioS)

Amyotrophic lateral sclerosis is a lethal neurodegenerative disease without any current treatment. Developing effective therapeutics will require improved understanding of the mechanisms underlying disease onset and progression. ALS/FTD-associated genes discovered in recent years suggest that in addition to inducing neuronal dysfunction, altered function of inflammatory cells in the brain and periphery could contribute directly to disease pathogenesis. However, despite extensive research on immune cells in the context of ALS, we are just beginning to decipher the exact nature of microglial activation states and heterogeneity, communication between different cell types at different stages of disease, and how combined microglial and neuronal dysfunction could drive neurodegeneration (Fig. 2).

It is notable that C9orf72, TBK1, OPTN, TARDBP and other ALS/FTD genes are highly expressed in innate immune cells, and several already have evidence linking their dysfunction to altered immunity. It will be interesting to determine if alterations in function of these genes lead to a consistently altered pattern of responses to pathogens and immune tolerance, which could potentially explain epidemiological associations with autoimmunity and cancer risk in ALS and FTD. Further research is needed to understand exactly which immune cells are driving this altered innate immune tone, and whether carriers of these genetic variants have definable altered peripheral immune responses, but this may open the door to novel therapeutic strategies and additional disease biomarkers.

Another key point is that an abnormal immune system is not itself presumably sufficient to cause motor neuron death, and that additional factors are clearly necessary to initiate disease. From this perspective, it is interesting to note that innate immune cells both in the brain and periphery are uniquely positioned to sense changes in environment (gut microbiome, infection, and traumatic injury) and communicate this to surrounding cells and, therefore, represent a key cell type where genetic variants could influence response to environmental factors to contribute to disease pathogenesis.
